# A Review of the Effects of Leucine Metabolite (β-Hydroxy-β-methylbutyrate) Supplementation and Resistance Training on Inflammatory Markers: A New Approach to Oxidative Stress and Cardiovascular Risk Factors

**DOI:** 10.3390/antiox7100148

**Published:** 2018-10-20

**Authors:** Hamid Arazi, Behzad Taati, Katsuhiko Suzuki

**Affiliations:** 1 Department of Exercise Physiology, Faculty of Sport Sciences, University of Guilan, Rasht 4199843653, Iran; taati.behzad@yahoo.com; 2 Faculty of Sport Sciences, Waseda University, Tokorozawa 359-1192, Japan; katsu.suzu@waseda.jp

**Keywords:** HMB, branched-chain amino acid, strength training, sports nutrition, inflammation

## Abstract

β-hydroxy β-methylbutyrate (HMB) is a bioactive metabolite formed from the breakdown of the branched-chain amino acid, leucine. Given the popularity of HMB supplements among different athletes, specifically, those who participate in regular resistance training, this review was performed to summarize current literature on some aspects of HMB supplementation that have received less attention. Because of the small number of published studies, it has not been possible to conclude the exact effects of HMB on cardiovascular parameters, oxidative stress, and inflammatory markers. Thus, the interpretation of outcomes should be taken cautiously. However, the data presented here suggest that acute HMB supplementation may attenuate the pro-inflammatory response following an intense bout of resistance exercise in athletes. Also, the available findings collectively indicate that chronic HMB consumption with resistance training does not improve cardiovascular risk factors and oxidative stress markers greater than resistance training alone. Taken together, there is clearly a need for further well-designed, long-term studies to support these findings and determine whether HMB supplementation affects the adaptations induced by resistance training associated with the body’s inflammatory condition, antioxidative defense system, and cardiovascular risk factors in humans.

## 1. Introduction

Some athletes believe that most normal diets do not provide sufficient demands for an optimum performance during intensive training and competitions. Dietary supplements are a commonly used strategy to improve exercise performance and recovery, and many athletes use them as a part of their regular training or competition routine [1]. Over the last decades, numerous studies have been conducted to identify anabolic nutrients for skeletal muscles. 

β-hydroxy β-methylbutyrate (HMB) is a type of amino acid supplement on the market. Due to its beneficial effects on muscle function and protein synthesis [2,3], HMB is fast becoming popular among different athletes as an ergogenic aid [4,5]. HMB is added to many training protocols, with the hopes of an enhanced lean body mass and sports performance [6]. Scientific research during the past 20 years demonstrate that HMB supplementation in conjunction with resistance training may improve body composition [7,8,9,10,11,12], muscle strength [2,7,9,10,11,12,13], and power [7,10,13]. It has also been reported that supplying HMB promotes favorable changes in aerobic [14,15,16] and anaerobic [11,14,17] capacity, and muscle recovery after exercise [12,18,19,20,21] in different athletes. To date, several systematic reviews and meta-analyses have investigated the aforementioned actions of HMB supplementation in a variety of populations [4,5,22,23,24,25,26]. Although recent studies have proposed some other actions of HMB with regard to effects on inflammatory, cardiovascular, and oxidative stress markers, these new aspects have received less attention. Therefore, we aimed to review recent findings in these contexts.

## 2. A Brief Overview of HMB Metabolism

**Figure 1:**
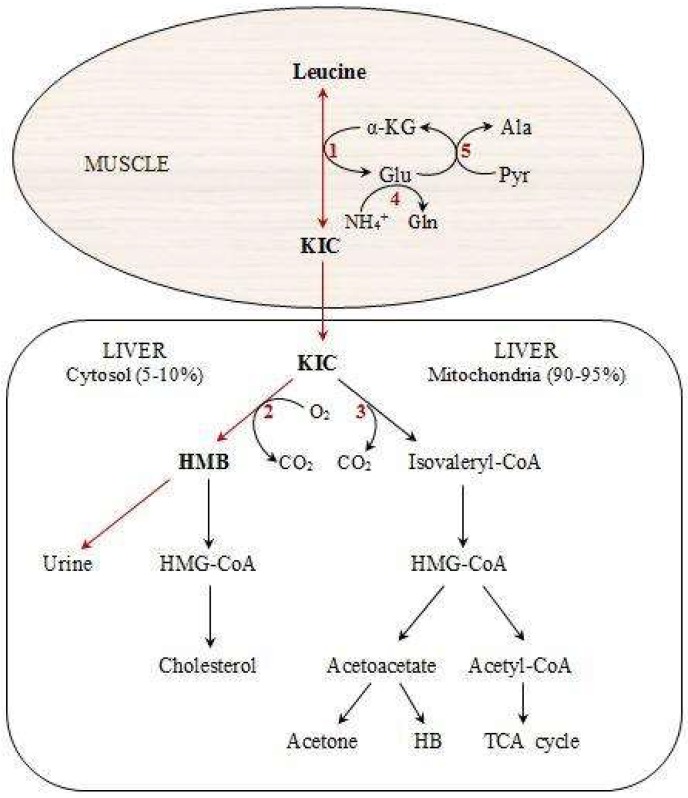
Pathways of HMB metabolism from the amino acid, leucine. Modified from Nissen and Abumrad [31]. HMB: Beta-hydroxy-beta-methylbutyrate, KIC: Alpha-ketoisocaproic acid, HB: Beta-hydroxybutyrate, HMG-CoA: 3-hydroxy-3-methyl-glutaryl-CoA, Ala: Alanine, Pyr: Pyruvate, 1: branched-chain amino acid (BCAA) aminotransferase, 2: KIC dioxygenase, 3: Branched-chain alpha-keto acid dehydrogenase (BCKAD), 4: Glutamine synthetase, 5: Alanine aminotransferase.

HMB is a metabolic by-product of the essential branched-chain amino acid (BCAA), leucine, which has key roles in protein metabolism [27]. Figure 1 shows the different steps in the production of HMB [25,28]. Following the reversible transamination of leucine to α-ketoisocaproate (KIC) through the enzymatic action of BCAA transferase [26], KIC in the liver can either produce isovaleryl-CoA by the enzyme, branched-chain ketoacid dehydrogenase [28], or generate HMB by the cytosolic enzyme, KIC dioxygenase [29]. Most amounts of produced KIC are metabolized into isovaleryl-CoA, and it has been estimated that only approximately 2–10% of leucine is oxidized into HMB [25,30]. The normal plasma range of HMB concentrations is 1 to 4 µmol/L, but can increase 5-to 10-fold following leucine administration [31]. Although some foods, including citrus fruits, some fish, and breast milk, have some HMB [28], it is impractical to provide the typical 3 g daily dosage of HMB used in most previous human studies that demonstrate an improvement of body composition [7,8,10,11,12] and muscle strength [2,7,10,11,12,13]. Therefore, HMB supplementation is a reasonable way for different athletes, specifically, those who participate in resistance training programs. 

There are two commercially available forms of HMB supplement, including (i) calcium HMB (HMB-Ca), a mono-hydrated calcium salt; and (ii) a free acid form of HMB (HMB-FA), beta-hydroxy-beta-methylbutyric acid [23,32]. Depending upon the dose and its ingestion with other additional nutrients, the magnitude and rate of appearance in blood circulation, and the clearance rate of HMB following consumption are different. In this context, Vukovich et al. [33] compared two doses of HMB-Ca and found that a 3-g dose can cause a peak in plasma concentrations of HMB 1 h after consumption, while a peak HMB level occurred 2 h after the ingestion of a 1-g dose. Plasma concentrations of HMB and urinary losses with the 3-g dose were also significantly higher than the 1-g dose (300% and 14%, respectively). The authors also reported that adding 75 g of glucose to the HMB-Ca dosage may delay peak HMB concentrations by 1 h and decrease its magnitude because of slow gastric emptying or an improvement in HMB clearance [33]. However, compared to 1 g of HMB-Ca, the absorption rate of 0.8 g of HMB-FA was higher and it only took 36 min to reach peak plasma concentrations following ingestion [32]. Furthermore, these increases in peak plasma levels were accompanied by a higher plasma clearance rate (25%) compared to HMB-Ca, indicating greater tissue uptake and utilization [23,25,34]. Although these benefits provided more effective and practical effects on muscle recovery after exercise through the greater intra-muscular HMB bioavailability [23,32,35], another study reported higher bioavailability of HMB after HMB-Ca intake when compared with an equivalent dosage of HMB-FA [36]. Taken together, most published studies have administered HMB-Ca, and further research is needed in this area. 

## 3. An Overview of Different HMB Effects and Its Potential Mechanisms

We have summarized a number of the most important and beneficial effects of HMB supplementation and its suggested mechanisms of action (Table 1).

## 4. Effects of HMB on Inflammation

### 4.1. Effects on Inflammation without Exercise

HMB improves immune function, especially under stressful conditions. In vitro, HMB has been shown to increase lymphocyte blastogenesis in a dose-dependent fashion [31]. In an animal study, Peterson et al. [48] found that HMB enhanced nitrite production in macrophages and also antibody production. The favorable effects of HMB supplementation on the number of CD3 and CD8 cells and human immunodeficiency virus (HIV) load have also been reported [49]. These data were supported by Hsieh et al. [50], who concluded that supplementation of HMB at 3 g/day for seven days may have an anti-inflammatory effect in a group of elderly patients with chronic obstructive pulmonary disease (COPD). 

### 4.2. Effects on Inflammation Following Exercise

It has been well documented that acute exercise, specifically high-intensity exercise, results in increased inflammatory markers [51]. However, regular exercise training exerts anti-inflammatory effects on the blood [52]. The investigation of new strategies for decreasing the body’s inflammatory reactions after exercise may provide further insight into improved recovery and subsequent performance. It has been suggested that some pro-inflammatory factors, such as interleukin (IL) 1 beta (IL-1β) and tumor necrosis factor alpha (TNF-α), increase proteolysis and may modulate protein turnover [53]. Because HMB is associated with less proteolysis [39,41], to date, several studies have focused on unraveling whether HMB supplementation affects the inflammatory responses following exercise training (Table 2). Beneficial effects of acute HMB supplementation on attenuating pro-inflammatory mediators have been reported in resistance-trained athletes [54,55]. Townsend et al. [54] showed that circulating pro-inflammatory markers, TNF-α and monocyte TNF-α receptor 1 (TNFR1), expression was elevated during an acute bout of heavy resistance exercise and subsequent recovery in healthy, resistance-trained men. However, HMB-FA supplementation (acute ingestion before and/or after exercise) decreased these mediators immediately after resistance exercise. However, another study did not support these findings. Additionally, in another well-designed study, acute HMB ingestion (30 min before, and 2 and 6 h after exercise) had no effect on the responses of macrophage inflammatory protein (MIP)-1β, but attenuated the significant peak expression of complement receptor type 3 (CR3) at 30 min post-exercise (four sets of up to 10 repetitions of three resistance exercises at 70–80% 1-repetition maximum). No increases in CR3 expression after exercise or during recovery was also observed by HMB. However, these changes did not contribute to a more rapid recovery or improve subsequent performance [55]. Longer duration of HMB supplementation may also have some beneficial effects on the inflammatory response after exercise. Hoffman et al. [56] revealed that 23 days of HMB supplementation in combat soldiers can attenuate the inflammatory response (TNF-α, IL-8, IL-10, granulocyte colony-stimulating factor, interferon-γ, and fractalkine) to intense military training, and maintain muscle quality.

Although these data provide evidence for a potential blunted or delayed inflammatory response following intense exercise protocols with acute or three weeks of HMB supplementation, anti-inflammatory effects of HMB were not confirmed by others that examined acute or long-term effects of HMB. For instance, Vulcan [57] investigated the effect of acute supplementation of HMB (before, or before and after exercise) on inflammatory responses following three sets of 50 eccentric leg extensions on each leg. It was observed that a decrease in serum concentration of IL-1 receptor antagonist (IL-1ra) and TNF-α for the placebo group was attenuated by HMB at 48-h and 72-h post exercise. In another study, it was investigated whether a longer period of HMB administration can influence inflammatory mediators in elite, national team level adolescent volleyball players. In this study, Portal et al. [17] found that seven-week consumption of HMB (3 g/day) did not change serum concentrations of IL-6 and IL-1ra during the early phase of the volleyball season. It should be considered that the training stimulus in this research was different from that performed by the four aforementioned studies [54,55,56,57]. Therefore, additional studies with long-term supplementation with HMB (at least 10 to 12 weeks) are needed to determine the impact of this dietary supplement on inflammatory responses to resistance exercise.

Because of limited scientific data regarding HMB effects on oxidative stress and cardiovascular parameters following resistance exercise, we propose new approaches for future research.

## 5. An Approach to Oxidative Stress

To the best of our knowledge, the available scientific literature on the effect of HMB supplementation on oxidative stress in humans is still preliminary in nature and it should be taken into more accurate consideration. Although HMB may improve immune function in humans [54,55,57], unfortunately, there is only one study [58] that has directly examined the effects of HMB supplementation on oxidative stress responses to exercise training (Table 2). In a randomized, double-blind, placebo-controlled trial, we investigated the effects of six-week HMB-FA supplementation on oxidative stress markers in 16 healthy young males. In this study, 8-hydroxy-2-deoxyguanosine (8-OHdG), malondialdehyde (MDA), and protein carbonyl (PC) were measured 48 h before and after resistance training. A significant decrease in MDA and PC was observed in both placebo and HMB groups. However, 8-OHdG did not change after resistance training in any of the groups. Thus, it seems that adding HMB supplementation to resistance training had no further improvements related to oxidative stress markers [58]. A transient increase in oxidative stress following acute exercise may be linked to the health benefits of regular exercise. Therefore, it is unknown what suppression of this oxidative response may do long-term with HMB supplementation. Given the limited available data about HMB effects on oxidative stress mediators, more research examining its effects is warranted. Only after more well-designed trials of HMB have been performed and its effects on the oxidative stress profile have been better defined will it be possible to comment on the effectiveness of HMB as a dietary supplement. 

## 6. An Approach to Cardiovascular Risk Factors

### 6.1. Cardiovascular Effects of HMB without Exercise

As shown in Figure 1, cytosolic β-hydroxy-β-methylglutarate-Co-A (HMG-CoA), which is produced from HMB in the cytosol of the liver, can be used for cholesterol synthesis [59]. Thus, HMB action as a precursor for cellular cholesterol synthesis can be important for membrane production during periods of high muscular stress, especially during exercise training and the subsequent recovery period. This is known as the cholesterol synthesis hypothesis (CSH) [60]. There are controversial findings about the effect of HMB on some cardiovascular risk factors. For example, different authors have found no change [61], an increase [62], or a decrease [60] in low-density lipoprotein (LDL) or total cholesterol (TC). In a comprehensive study, Nissen et al. [60] summarized data from nine studies in which humans were fed 3 g HMB/day for three to eight weeks and reported that HMB supplementation results in a net decrease in TC (5.8%), a decrease in LDL cholesterol (7.3%), and a decrease in systolic blood pressure (4.4 mmHg). However, Hsieh et al. [61] concluded that HMB supplementation (2 g/day) for two or four weeks has no significant effect on serum lipids in bed-ridden elderly men and women. More recently, an animal study examined the effectiveness of a 12-week HMB administration on insulin resistance induced by a high fructose diet in rats. Compared to the control group, HMB significantly enhanced insulin tolerance and decreased fasting insulin, insulin resistance index (HOMA-IR), glycosylated hemoglobin (Hb_A1c_), hepatic glycogen content, and serum triglycerides (TG), LDL, and very low-density lipoprotein (VLDL) [63]. It has also been demonstrated that two-week supplementation with HMB reduces arrhythmias during ischemia induced in rats [64]. Generally, these data suggest that HMB supplementation may result in a decrease in the risk of heart attack and stroke.

### 6.2. Cardiovascular Effects of HMB Following Exercise

Only one study investigated the cardiovascular effects of HMB supplementation in conjunction with exercise training (Table 2). Arazi et al. [65] examined the effect of HMB-Ca (3 g/day) on cardiovascular risk factors after four weeks of resistance training (three sessions per week) in athletes. After the training period, TC, LDL, and TG were significantly decreased in both groups and diastolic blood pressure was reduced only in the HMB group. However, no significant differences were found between HMB and placebo groups. Because of limited data regarding this topic, it is difficult to declare the certain and exact cardiovascular effects of HMB supplementation when combined with exercise training. Thus, further prolonged investigations are needed to determine these effects. 

## 7. Conclusions

The available data collectively indicate that acute ingestion of HMB before and after resistance exercise can attenuate some circulating pro-inflammatory mediators, which improves the subsequent recovery process. However, more research is needed to support these effects and verify if chronic HMB consumption in conjunction with resistance training has more favorable effects on pro- and anti-inflammatory markers. Although the number of studies examining the interaction effects of HMB and exercise training on inflammation, oxidative stress, and cardiovascular parameters are limited, it seems that adding HMB supplements to a resistance exercise protocol did not produce further benefits. Generally, future research should be performed to specify the effectiveness of HMB supplementation on the inflammatory profile, the body’s antioxidative defense system and oxidative stress markers, and cardiovascular risk factors, when combined with exercise training.

## Figures and Tables

**Table 1:** A summary of potential mechanisms proposed for the different beneficial effects of HMB supplementation on skeletal muscle.

Effect	Mechanisms of action
Increasing protein synthesis	Stimulation of the mammalian target of rapamycin (mTOR) signalling pathway: Inhibition of MuRF-1 expression, and phosphorylation of FoxO1 and FoxO3a through activation of the PI3K/Akt signalling pathway [3]; increasing the expression of MyoD and MEF2, activation of the MAPK/ERK and PI3K/Akt pathways, leading to myogenic cell proliferation [37]. Stimulation of the GH/IGF-1 axis: Increasing the mRNA expression of pituitary GH and serum concentrations of IGF-1 [38].
Decreasing proteolysis (protein breakdown)	Down-regulation of catabolic signalling pathways, including ubiquitin-proteasome and autophagy-lysosome systems: Inhibiting proteasome expression, reducing activities of proteasome enzyme, down-regulation of caspases, decreasing the apoptosis of myonuclei [39,40,41,42]. Increasing GH and IGF-1 [43].
Enhancing tissue repair	Increasing proliferation of satellite cells [37]. Increasing cholesterol synthesis: HMB acts as a substantial precursor of cell membranes and improves the repair of sarcolemma after contractile activity [44].
Improving excitation-contraction coupling in muscle cells	Increasing calcium release from the sarcoplasmic reticulum (SR) [45].
Improving aerobic capacity	Increasing mitochondrial biogenesis and fat oxidation [46]. Increasing the hormone-sensitive lipase (HSL) gene and protein expression in white adipose tissue because of increased GH levels, leading to increased lipolysis, and thus, more lipid availability [28,38].
Delaying acute muscle fatigue	Increasing the content of mitochondrial acetyl-CoA through the conversion of HMG-CoA into acetoacetyl-CoA [30,31].
Increasing ATP and glycogen content in skeletal muscle	Accelerating the TCA cycle, increasing malate-aspartate shuttle, and providing needed carbon for glycogen synthesis [47].

**Table 2:** Main characteristics of studies examining the effects of HMB supplementation on inflammatory, cardiovascular, and oxidative stress markers.

Author (Year)	Subjects	Exercise Protocol	Supplementation	Primary Findings	Conclusion
Townsend (2013) [54]	40 resistance-trained men	4 sets of the squat (80% of 1RM), and dead lift and barbell split-squat (70% of 1RM); as many repetitions as possible (up to 10 repetitions for each set); 90 s rest intervals between sets and exercises	3 g (1-g serving of HMB-FA, 30 min before, and 2 h and 6 h following the exercise session)	↑ TNF-α from pre to immediately post-exercise in only PL group; ↑ TNFR1 expression from pre to 30 min post-exercise in only PL group	Acute HMB-FA supplementation may attenuate the initial immune responses to an intense RE, which may reduce subsequent recovery period
Vulcan (2012) [57]	Untrained subjects (16 men, 16 women)	3 sets of 50 eccentric leg extensions from 0° to 90° at a rate of 60°/s on both legs; 2 min rest intervals between sets	Pre-exercise, or pre-exercise and for 4 days (3 servings/day) post-exercise; either HMB-Ca or HMB-FA	↑ IL-1ra at 48 h, 72 h, and 96 h post-exercise compared to PL; ↑ TNF-α at 48 h, and 72 h post-exercise compared to PL	The role of acute HMB supplementation on reducing the inflammatory response after RE has not been confirmed
Gonzalez (2014) [55]	39 resistance-trained men	4 sets of the squat, dead lift and barbell split-squat (70–80% of 1RM); as many repetitions as possible (not to exceed 10 repetitions in any set); 90 s rest intervals between sets and exercises	3 g (1-g serving of HMB-FA, 30 min before, and 2 h and 6 h following the exercise session)	↔ MIP-1β responses; ↓ peak expression of CR3 at 30 min post-exercise; ↑ percentage of monocytes expressing CR3 for up to 48 h post-exercise	HMB supplementation may alter immune cell mobilization and adhesion mechanisms during tissue recovery after RE
Hoffman (2016) [56]	11 elite combat male soldiers	A combination of different military training including combat skill development, extreme trainings, navigational training with carrying approximately 35 kg of equipment	3 servings (1 g/serving) of HMB-FA per day at meal time for 23 days	↓ TNF-α responses; ↓ G-CSF; ↓ IL-10; ↓ INFγ; ↓ IL-8; ↓ CX3CL1 from pre- to post-training	HMB supplementation may attenuate the inflammatory mediators to severe military trainings, and maintain muscle quality
Portal (2011) [17]	Adolescent elite volleyball players (14 males, 14 females)	Volleyball training (the early phase of the volleyball season)	3 g/day HMB supplementation for 7 weeks; the type of HMB has not been mentioned	↔ IL-10 ↔ IL-1ra from pre- to post-training	HMB supplementation has no significant effect on the inflammatory mediator changes during the initial phases of volleyball training season
Arazi (2018) [58]	16 healthy young males	3 sets of 8–12 repetitions with 75–85% of 1RM (leg press, knee extension, knee flexion, lat pull-down, bench press, shoulder press, cable biceps curl and triceps push down); 2 sessions/week for 6 weeks; 2 and 3 min rest intervals between sets and exercises, respectively	3 g/day HMB-FA; one Serving (1 g) with each of 3 separate meals	↓ MDA and PC from pre- to post-training in both HMB and PL groups; ↔ 8-OHdG in the groups	HMB supplementation has no further improvements related to oxidative stress markers in young males
Arazi (2015) [65]	20 male athletes (without regular resistance training)	2 sets of 9 exercises (squat, knee extension, knee flexion, leg press, bench press, lat pull- down, shoulder press, cable biceps curl, and triceps push down); 10 repetitions with 80–85% of 1RM; 2 and 3 min rest intervals between sets and exercises, respectively	3 g/day HMB-Ca; 3 servings (1 g) per day	↓ TC, LDL, and TG from pre- to post-training in both HMB and PL groups; ↓ systolic BP and ↑ HDL in HMB group; ↔ diastolic BP, RBC, Hb, Hct, MCV, and MCH in the groups	HMB supplementation is safe and does not result in any adverse effects on cardiovascular parameters in male athletes

↑ increase, ↓ decrease, ↔ no change, PL: Placebo, RE: Resistance Exercise, 1RM: 1-repetition maximum, IL-1ra: IL-1 receptor antagonist, MIP: Macrophage inflammatory protein, CR3: Complement receptor type 3, G-CSF: Granulocyte colony-stimulating factor, INFγ: Interferon-γ, CX3CL1: Fractalkine, 8-OHdG: 8-hydroxy-2-deoxyguanosine, MDA: Malondialdehyde, PC: Protein carbonyl, TC: Total cholesterol, TG: Triglycerides, LDL: Low-density lipoproteins, HDL: High-density lipoproteins, BP: Blood pressure, RBC: Red blood cells, Hb: Hemoglobin, Hct: Hematocrit, MCV: Mean corpuscular volume, MCH: Mean corpuscular hemoglobin.
